# Skeletal muscle fibre type and enzymatic activity in adult offspring following placental and peripheral malaria exposure in foetal life

**DOI:** 10.3389/fpubh.2023.1122393

**Published:** 2023-06-02

**Authors:** Dirk L. Christensen, Theonest K. Mutabingwa, Ib C. Bygbjerg, Allan A. Vaag, Louise G. Grunnet, Fanny Lajeunesse-Trempe, Jannie Nielsen, Christentze Schmiegelow, Kaushik L. Ramaiya, Kathryn H. Myburgh

**Affiliations:** ^1^Department of Public Health, University of Copenhagen, Copenhagen, Denmark; ^2^Faculty of Medicine, Hubert Kairuki Memorial University, Dar es Salaam, Tanzania; ^3^Department of Immunology and Microbiology, University of Copenhagen, Copenhagen, Denmark; ^4^Department of Clinical Sciences, Lund University, Lund, Sweden; ^5^Translational Type 2 Diabetes Research, Steno Diabetes Center Copenhagen, Herlev, Denmark; ^6^Clinical Prevention Research, Steno Diabetes Center Copenhagen, Herlev, Denmark; ^7^Faculty of Medicine, Laval University, Quebec, QC, Canada; ^8^Shree Hindu Mandal Hospital, Dar es Salaam, Tanzania; ^9^Department of Physiological Sciences, Stellenbosch University, Stellenbosch, South Africa

**Keywords:** malaria exposure, hypoxia, myosin heavy chain, skeletal muscle enzymes, glucose metabolism

## Abstract

**Background:**

Maternal malaria may restrict foetal growth. Impaired utero-placental blood flow due to malaria infection may cause hypoxia-induced altered skeletal muscle fibre type distribution in the offspring, which may contribute to insulin resistance and impaired glucose metabolism. This study assessed muscle fibre distribution 20 years after placental and/or peripheral *in-utero* malaria exposure compared to no exposure, i.e., PPM+, PM+, and M-, respectively.

**Methods:**

We traced 101 men and women offspring of mothers who participated in a malaria chemosuppression study in Muheza, Tanzania. Of 76 eligible participants, 50 individuals (29 men and 21 women) had skeletal muscle biopsy taken from *m*. vastus lateralis in the right leg. As previously reported, fasting and 30 min post-oral glucose challenge plasma glucose values were higher, and insulin secretion disposition index was lower, in the PPM+ group. Aerobic capacity (fitness) was estimated by an indirect VO_2_max test on a stationary bicycle. Muscle fibre sub-type (myosin heavy chain, MHC) distribution was analysed, as were muscle enzyme activities (citrate synthase (CS), 3-hydroxyacyl-CoA dehydrogenase, myophosphorylase, phosphofructokinase, lactate dehydrogenase, and creatine kinase activities. Between-group analyses were adjusted for MHC-I %.

**Results:**

No differences in aerobic capacity were found between groups. Despite subtle elevations of plasma glucose levels in the PPM+ group, there was no difference in MHC sub-types or muscle enzymatic activities between the malaria-exposed and non-exposed groups.

**Conclusion:**

The current study did not show differences in MHC towards glycolytic sub-types or enzymatic activity across the sub-groups. The results support the notion of the mild elevations of plasma glucose levels in people exposed to placental malaria in pregnancy being due to compromised pancreatic insulin secretion rather than insulin resistance.

## Introduction

Several studies over the past seven decades have shown foetal growth restriction due to malaria exposure (1–3). Globally, there are an estimated 50 million annual pregnancies in high-endemic malarious areas of which at least 25% of pregnant women are being infected with malaria (4). The mechanism(s) behind foetal growth restriction and its relationship with malaria exposure is not fully understood, but based on Doppler-scans it has been shown that blood flow from the placenta to the foetus is compromised (5). Furthermore, malaria infection in early pregnancy has been shown to impede placental vascular development (6). These changes will cause restricted oxygen delivery to the foetus, which according to histological examination of within-term placenta is due to impaired placental function by malaria parasitic infiltration of the villi (7).

Skeletal muscle is an essential tissue for movement and plays a major role in whole body metabolism. The substrates supplying the energy required for contraction are high-energy phosphates, carbohydrate, lipid or protein (8, 9). Skeletal glucose uptake is stimulated by insulin (10–12), and further enhanced by the intracellular calcium increases accompanying muscle activation-contraction coupling (13). During exercise, when energetic demand is high, blood glucose can account for approximately 40% of blood glucose disposal (14). At rest, insulin signalling plays the predominant role. Indeed, in a classical study using the euglycaemic insulin clamp technique in healthy young men at rest, it was found that skeletal muscle contributes to approximately 85% of the glucose clearance (15). This is reduced by 50% in individuals with type 2 diabetes mellitus (DM) (15).

The contractile and metabolic properties in combination provide muscle fibre phenotypes. The major fibre types, namely fast and slow twitch fibres have characteristic contractile speed, as a result of specific myosin heavy chain (MHC) isoforms (16). The fibre type metabolic profiles are typically described as glycolytic or oxidative (17), characteristics that can be assessed in muscle samples by determining enzymatic capacity of key enzymes. Type I muscle fibres contain MHC-I, and oxidative enzyme activity is high, whereas type II muscle fibres contain MHC-II and are more glycolytic in nature. Although the proportion between these fibre types is heritable (18), a high level of exercise training can cause shifts in fibre type (19). Type II fibres have sub-types containing MHC-IIx, which are more glycolytic and MHC-IIa, which are more oxidative and therefore metabolically adjusted for energy utilisation, including oxidation of glucose. Studies of rat muscle with predominantly Type I highly oxidative fibres have higher protein levels of insulin receptors and glucose transporters reflecting their capacity to use glucose as substrate (20, 21). In addition, a functional study in rats in which insulin resistance was induced by high-fat diet, showed that insulin-stimulated glucose uptake into muscle was lower and that this was driven by lower uptake in type II fibres specifically (22).

Skeletal muscle is adaptable to environmental stimuli such as altitude (23). Populations living at high altitude have lifelong exposure to hypoxia, which influences their skeletal muscle (24). Lifelong high altitude living shifts oxidative metabolism to a more glycolytic metabolism (25). Pathological conditions can also change muscle fibre type proportions. Patients with chronic obstructive pulmonary disease have higher type II fibre proportion (26), and lower oxidative enzyme activities (27). In obese middle-aged men and women with type 2 DM, proteomic analysis of muscle biopsies revealed higher fast muscle associated proteins and glycolytic pathway proteins, alongside lower levels of proteins associated with the slow and oxidative phenotype (28). Even in glucose-intolerant persons with moderately elevated fasting glucose, fibre type profile was skewed to higher fast twitch glycolytic type II fibres (29). Foetal development may be influenced during pregnancy at high altitude, and this has been shown particularly with regard to low birth weight (LBW) (30). Furthermore, LBW (a proxy for intrauterine growth restriction) is known to be associated with a fibre type profile towards higher proportion of MHC-IIx containing fast glycolytic fibres at the expense of the more oxidative MHC-IIa containing fibres (31).

We, among others (32), have previously hypothesized that a physiological adaptation to maternal malaria exposure takes place through epigenetic changes and foetal programming with focus on skeletal muscle among others as peripheral insulin resistance is one of the hallmarks of type 2 DM (33). From a clinical perspective and based on standard oral glucose tolerance test (OGTT) in the same cohort, we recently demonstrated subtle elevated plasma glucose 30 min post glucose ingestion in adult offspring of women exposed to peripheral and placental malaria in pregnancy, suggesting an early risk marker for later development of type 2 DM (34).

We aimed to determine whether exposure to combined peripheral and placental malaria compared to peripheral malaria exposure only, or no malaria, in the foetal state had an influence on skeletal muscle fibre distribution, and/or on oxidative or glycolytic skeletal muscle enzymatic activities in young adults.

## Materials and methods

We retrieved information from study documents on offspring and their mothers from a study on malaria chemosupression conducted in 1989–92 in Muheza, Tanga region, north-eastern Tanzania (35). During the original chemosupression study, pregnant women were given malaria chemoprophylaxis/chemosupressive regimens, and ensuing clinical malaria attacks were treated with sulphadoxine–pyrimethamine and if failed with quinine. For the current study, we retrieved relevant data including birth weight, mother’s age at birth, and maximum peripheral malaria density and frequency of malaria attack during pregnancy. During pregnancy, malaria exposure was determined by examining Giemsa stained thick blood smear taken every 2 weeks for peripheral malaria, and used a similar approach for a placental thick blood smear at delivery (35).

This follow-up study was conducted in 2010–11, and clinical results have been described (34). Consenting participants went through pre-study HIV counselling, and were then subjected to a rapid HIV (capillus) test, hepatitis B (Hb-surface antigen) test, and peripheral blood smear for malaria microscopy. A positive HIV or hepatitis B test were exclusion criteria, while a positive malaria test resulted in postponement of eligibility to participate in the study until the infection had been cleared. Participants who met the study inclusion criteria were instructed to abstain from alcohol, smoking, and heavy physical activity for at least 72 h prior to enrolment into the study.

We stratified study participants by foetal malaria exposure as follows: (1) both peripheral and placental malaria (PPM+), (2) peripheral malaria (PM+) at least once during pregnancy, and (3) peripheral and placental malaria negative (M-). LBW was defined as <2.5 kg.

Following an overnight fast, standard anthropometric measurements including height (m), weight (kg), and waist circumference (cm) were measured, and body mass index (BMI, kg/m^2^) was calculated. Body fat % and fat-free mass (FFM) were estimated using bio-impedance analysis (Tanita SC 330-S, Tokyo, Japan). Furthermore, abdominal fat distribution was measured using ultrasound scanning technique (Aquila Basic Unit, Esaote, Pie Medical Equipment) (36). Blood pressure (mmHg) and heart rate (beats/min) were measured three times after every 2 min using a full-automatic device (Omron HEM-7120, Kyoto, Japan) with the study participant seated. An average of the two last readings were used to calculate mean values. Baseline fasting blood glucose was measured, followed by a standard 75 g OGTT and 10 mL of blood drawn at 0, 30 and 120 min. Fasting, 30 min, and 120 min insulin EDTA-plasma insulin and C-peptide (pmol/L) were analysed according to the ECLIA-photon count method (Roche diagnostics). Insulin indices were calculated. A standard lipid profile was analysed by enzymatic identification-absorption photometry (Roche Cobas diagnostics). High-sensitivity C-reactive protein (hs-CRP) was analysed according to the immunoturbidimetric method (Roche Cobas diagnostics). Alanine transaminase aspartate transaminase, and creatinine were analysed via connected enzyme reactive method (Roche Cobas diagnostics); carbamide was analysed according to HPLC method (Dionex Ultimate 3,000, Thermo Scientific); gamma-Glutamyl transferase was analysed using the enzymatic calorimetric method (Roche Cobas diagnostics); Albumin was analysed by absorption photometry (Roche Cobas diagnostics). Fatty liver index was calculated according to a standard formula (37).

Maximal aerobic work capacity (watt-max method) was assessed using a Monark 828E stationary bicycle (Monark Exercise, Vansbro, Sweden), and calculated as VO_2_/min/kg according to the formula by (38). In brief, resistance was set at 0.75 and 1.0 kiloponds (kp) and the cadence was fixed at 60 and 70 rounds per minute for women and men, respectively, for 2–3 min of warm-up. Kp × cadence = watts. Immediately following warm-up, all participants started the test at 1.0 kp at the sex-specific cadence, i.e., 60 and 70 watts for women and men, respectively. Resistance was increased by 0.5 kp every 90 s until exhaustion, i.e., when an individual was unable to keep up the cadence. Maximal aerobic work capacity was then predicted from the formula: 0.16 + (0.0117 × maximal power output in watts) (38).

### Myosin heavy chain composition and enzyme activities in muscle homogenate samples

A fasting biopsy was taken from the right *m.* vastus lateralis using the Bergström needle technique with suction (39), and frozen in liquid nitrogen. A small piece of muscle was quickly weighed, transferred to a pre-cooled glass tube and a volume of chilled 100 mM potassium phosphate buffer, pH 7.30 added to a ratio of 1 mg:100 μL. Samples were kept on ice, homogenised using a glass homogeniser and sonicated (Virsonic Virtis) three times for 10 s on ice, with 10 s delay between intervals. The protein content of each sample was determined using the method described by (40).

**Figure 1:**
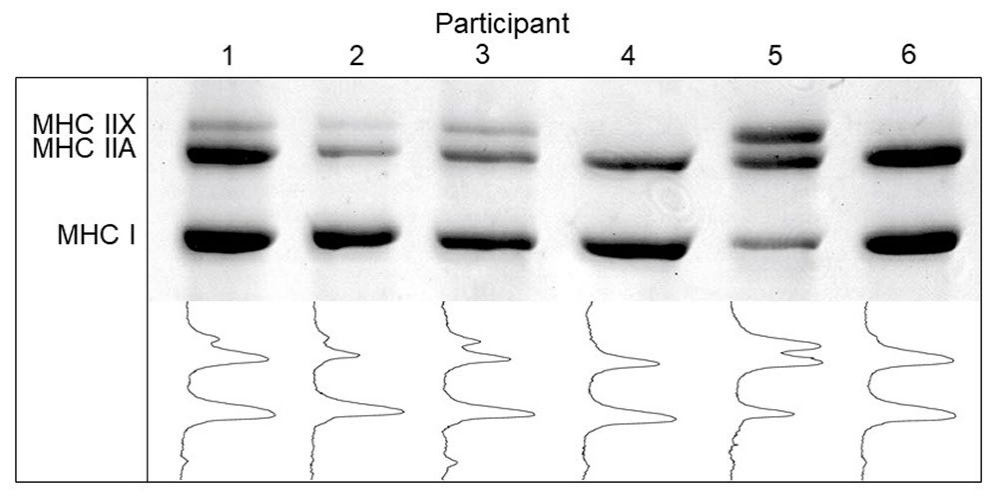
Myosin heavy chain (MHC) isoform content and densitometric profile of skeletal muscle biopsies in young Tanzanian adults (*n*=50).

MHC isoform contents of homogenate samples were determined using SDS-PAGE according to the method described by (41) with slight modifications. Briefly, electrophoresis was carried out at 4°C using a large-gel system (Hoefer SE600), first at constant 70 V for 4 h, followed by constant 275 V for 20 h. Gels were stained with Coomassie blue R250 and subsequently scanned using a computer scanner. Relative percentages of the bands were quantified using the Un-Scan-It software package ver 6.3 (Silk Scientific Corporation, Orem, UT, United States). See Figure 1 for images of the scanned Coomassie stained gels for measurement of muscle fibre types.

Citrate synthase (CS), 3-hydroxyacyl-CoA dehydrogenase (3-HAD), myophosphorylase (PHOS), phosphofructokinase (PFK), lactate dehydrogenase (LDH), and creatine kinase (CK) activities were determined using microplate fluorometric (SpectraMax id3, Molecular Devices, San Jose, CA, United States) adapted methods as described by (42). The enzyme reagent for all assays was 250 μL and sample volumes for CS, 3-HAD, PHOS, and PFK were 5 μL and 2 μL for the LDH assay. The original homogenate was further diluted (5×) as a volume of 2 μL used for the CK assay. The emission at 460 nm was recorded for 5 min with 30 s intervals using an excitation wavelength of 340 nm. A NADH or NADPH standard curve was included to calculate enzyme activities expressed as μmoles NADH or NADPH per minute per gram protein (μmol/min/g wet weight).

### Statistical analyses

Linear regression analyses were used to test for differences in outcomes of interest and malaria exposure. Furthermore, all regression analyses for the clinical data were adjusted for MHC-I % due to skewed sex distribution, and as we sought to elucidate if this may be related to intrinsic skeletal muscle phenotype. Regression analyses for muscle enzyme activity were adjusted for fitness expressed as estimated VO_2_max/min/kg fat-free mass. Estimated fitness was expressed as VO_2_/min/FFM to further account for the skewed sex distribution in the three study groups. Differences in LBW proportion between groups were determined by Fisher’s exact test. Continuous data are presented as mean (standard deviation, SD) and as median (interquartile range, IQR) for skewed number distribution. All analyses were performed using Stata 17.0 (*Stata* Statistical Software, College Station, *TX*: *StataCorp* LLC) with *p*-values <0.05 considered as statistically significant.

## Results

We traced 101 out of 337 mothers who participated in the original study, from whom 76 offspring were eligible after pre-study screening procedure. Biopsy samples were taken from 50 individuals (13 in the M-group, 28 in the PM+ group, and 9 in the PPM+ group).

Sex distribution was 21 women and 29 men with a mean age of 19.6 (0.9) years. There was no difference in proportion of LBW between the groups [(n = 1 (7.7%), n = 7 (25.0%), and n = 4 (7.44.4%) for M-, PM+, and PPM+, respectively, p = 0.15)]. Background and clinical characteristics of the mothers (while pregnant) are presented in Table 1. Furthermore, continuous variables of the offspring with inter-group differences are presented in Table 1 and Supplementary Table S1. Mean fasting glucose was highest in the PPM+ group (*p* = 0.074), and based on the OGTT, mean plasma glucose was highest in the PPM+ group at 30 min (*p* = 0.049) sustaining already published results (34). Disposition index (early phase insulin secretion/homoeostasis model assessment of insulin resistance) was lowest in the PPM+ group (*p* = 0.02).

**Table 1:** Background and clinical characteristics in offspring stratified by malaria exposure during pregnancy presented as mean (SD) (*n* = 50).

	Malaria negative	Peripheral malaria	Peripheral + placental malaria	*p*-value	*p*-value adjusted^a^
*N*	13	28	9		
Sex (male/female) (*n*)	8/5	13/15	8/1	0.08	
Mother’s age (year)	28 (8)	25 (7)	23 (5)	0.21	
Max malaria density in pregnancy (f/cc) peripheral	0	2,160 (800–13,400)	10,560 (3320–36,480)	0.19	
Attack frequency	0	2 (1–3)	2 (1–3)	0.88	
*Offspring*					
Birth weight (kg)	2.9 (0.6)	2.8 (0.5)	2.6 (0.5)	0.35	
Age (years)	20 (19–20)	20 (19–20)	20 (19–20)	0.95	
Height (cm)	1.62 (0.1)	1.61 (0.1)	1.62 (0.1)	0.93	0.89
Weight (kg)	52.4 (7.8)	51.9 (6.9)	52.3 (9.2)	0.97	0.96
Body mass index (kg/m^2^)	20.1 (2.7)	20.0 (2.1)	19.8 (2.7)	0.95	0.80
Fat (%)	11.0 (8.9)	13.0 (7.2)	8.6 (4.9)	0.28	0.64
Fat free mass (kg)					
Waist circumference (cm)	70.4 (6.5)	71.1 (5.4)	73.0 (6.0)	0.61	0.35
VAT (cm)^b^	5.4 (0.7)	5.3 (1.0)	5.7 (0.7)	0.52	0.58
SAT (cm)^c^	1.2 (0.4)	1.2 (0.6)	1.2 (0.4)	0.98	0.88
Aerobic fitness^d^ (mLO_2_/min/kg)	38.2 (10.2)	33.0 (13.6)	43.9 (11.9)	0.11	0.97
Aerobic fitness^d^^,^^e^ (mLO_2_/min/kg FFM)	42.7 (9.4)	37.3 (13.4)	47.6 (11.5)	0.11	
Fasting glucose (mmol/L)	4.5 (0.7)	4.6 (0.5)	5.0 (0.6)	0.09	0.074
Glucose 30 min (mmol/L)	6.8 (1.3)	6.7 (1.1)	8.2 (1.7)^b^	0.02	0.049
Glucose 120 min (mmol/L)	6.3 (1.0)	6.4 (1.3)	7.0 (2.6)	0.52	0.29
Fasting insulin (pmol/L)*	48 (29;84)	56 (48;87)	65 (39;70)	0.52	0.15
Insulin 30 min (pmol/L)*	465 (174;673)	224 (99;414)	233 (81;340)	0.50	0.84
Insulin 120 min (pmol/L)*	267 (189;452)	287 (205;480)	199 (101;228)	0.11	0.26
HOMA-IR^f^	1.59 (1.34)	2.03 (1.12)	1.72 (0.50)	0.64	0.64
Early phase insulin secretion (pmol/L)^g^	1,308 (598)	1,240 (644)	871 (330)	0.24	0.25
Disposition index^*,^^h^	1,290 (610;2,978)	615 (508;748)	527 (325;890)^a^	0.01	0.01
Matsuda index^*,^^i^	23.9 (12.3;55.2)	15.9 (12.5;20.6)	22.0 (16.1;31.1)	0.31	0.31

a Adjusted for myosin heavy chain-I percentage.

b VAT, visceral adipose tissue.

c SAT, subcutaneous adipose tissue.

d *n* = 45.

e Estimated fitness expressed relative to FFM, fat free mass.

f HOMA-IR, homeostatic model assessment of insulin resistance, formula: insulin 0 min*(glucose 0 min/22.5)*0.144.

g Early phase insulin secretion, formula: 1283 + 1.829*insulin 30 min − 138.7*glucose 30 min + 3.772*insulin 0 min.

h Disposition index, early phase insulin secretion/HOMA-IR.

i Matsuda index, formula: 10000*(√(glucose 0 min*insulin 0 min*mean glucose*mean insulin))^−1^; ^*^log transformed for value of *p* and numbers presented as geometric mean.

Two participants (1 M-, 1 PM+) were overweight by both BMI and central obesity criteria. Estimated fitness did not differ between groups in mean, or spread around the mean, when adjusted for sex. Two participants had hyperglycaemia, 1 DM, and 1 impaired fasting glucose, and both were from the PPM+ group. Two participants had C-peptide levels <300 pmol/L, both from the PPM+ group, and including the participant who had DM.

Skeletal muscle fibre type and enzymatic activities are presented in Table 2. No significant impact of malaria exposure on fibre type and any enzymatic activity were found either before or after adjustment for estimated fitness/min/kg FFM. The correlation between muscle enzyme activities and aerobic estimated fitness (VO_2_/min/kg) levels was significant for CS activity at (0.04 (95% CI: 0.01; 0.07, *p* < 0.005).

**Table 2:** Skeletal muscle (m vastus lateralis) fibre type distribution and enzymatic activity presented as mean ± SD (*n* = 50).

	Malaria negative	Peripheral malaria	Peripheral + placental malaria	*p*-value	*p*-value adjusted^a^
*N*	13	28	9		
*Electrophoresis (MHC)*^b^					
MHC-I (%)	53.5 ± 12.3	50.1 ± 11.1	55.8 ± 8.2	0.35	
MHC-IIa (%)	36.4 ± 6.2	40.0 ± 8.4	38.5 ± 6.1	0.44	
MHC-IIx (%)	8.0 ± 12.2	9.9 ± 9.0	7.8 ± 8.1	0.79	
*Enzymes*^c^					
Citrate synthase	5.4 ± 1.3	4.8 ± 1.3	6.0 ± 0.83	0.03	0.54
3-HAD^d^	5.4 ± 0.81	4.5 ± 1.0	5.4 ± 0.73	0.01	0.63
Myophosphorylase	15.1 ± 3.3	14.4 ± 4.1	17.4 ± 2.0	0.12	0.38
Phosphofructokinase	21.9 ± 6.7	20.1 ± 6.3	21.8 ± 5.9	0.63	0.78
Lactate dehydrogenase	35.8 ± 7.6	30.6 ± 7.9	37.3 ± 5.1	0.03	0.82
Creatine kinase	466 ± 75	415 ± 96	496 ± 43	0.03	0.70

a Adjusted for fitness as expressed as estimated VO_2_max/min/kg fat-free mass.

b Myosin heavy chain.

c μmol/min/g wet weight.

d 3-hydroxyacyl-CoA dehydrogenase.

MHC-I distribution was significantly different between women and men, with women having 47.6 (10.7)% and men 55.3 (10.2)%, respectively (*p* = 0.014).

## Discussion

The study neither found any impact of foetal malaria exposure on MHC distribution, nor on skeletal muscle enzyme activities, with or without adjustments for estimated fitness expressed by FFM. As this is the first attempt at documenting the effect of combined peripheral and placental malaria on skeletal muscle and their enzymatic activity in adult offspring, direct comparison with other studies is not possible. Nevertheless, increased metabolic demand is a feature of pregnancy (43), particularly in the second and third trimesters (44). This suggests that skeletal muscle along with the other organs of the offspring of mothers with malaria may have experienced chronic hypoxic insults – induced by anaemia – which could have led to adaptations such as a change in skeletal muscle with a preference for glycolysis over oxidative metabolism. This is characteristic of the fast twitch muscle (MHC-IIa and IIx) phenotypes, and here we hypothesized that such intrauterine phenotype change would still be evident in adulthood.

There could be two possible reasons for our negative findings on the main outcome variables of this study. First, malaria exposure, and thereby a state of hypoxia in the foetus, may not have been long enough to have had a negative impact. Active malaria attack frequency (duration of “active” malaria) was 1–3 weeks in the PPM+ group. Second, malaria prophylaxis blunted the impact of malaria parasitic infection, which could be another reason for the lack of impact on skeletal muscle and enzymatic activity. Furthermore, the negative impact on birth weight due to combined peripheral and placental malaria may have been counteracted due to the use of malaria prophylaxis.

Interestingly, there was a significant difference in the distribution of MHC-I between women and men. The literature is not consistent with regard to the possibility that women have less type I fibre proportion, as we have found. The current data is in agreement with Komi and Karlsson (45), but contrary to the findings of (46), both of which were studies in non-African populations.

In line with our recent study from a slightly larger group of study participants (*n* = 76) (34), we found higher plasma glucose after 30 min after glucose ingestion, but this time also a higher fasting plasma glucose level in the PPM+ group. Furthermore, disposition index (early phase insulin secretion/homoeostasis model assessment of insulin resistance) was significantly lower in the PPM+ group compared to the M-group. Plasma glucose elevations could arise from either insulin resistance or impaired pancreatic insulin secretion, with skeletal muscle being the major tissue involved in insulin stimulated peripheral glucose uptake, and thus also insulin resistance (15). Cross-sectional comparison between obese individuals and obese individuals with type 2 DM found a lower type I fibre proportion in those with type 2 DM (47); thus alterations of skeletal muscle fibre type compositions and/or enzymatic activities, might have contributed to insulin resistance in the offspring exposed to foetal malaria. Previous research has associated LBW with skeletal muscle characteristics in adulthood and the risk of developing insulin resistance (31). The young adults in the study with LBW did not differ from age-matched controls in Type I or type II fibre proportions, but considering the assessment of the subgroups of type II fibres, the LBW group had lower type IIa compared to type IIx fibres. As mentioned in the introduction, muscles with high oxidative metabolism have better capacity to take up glucose (21), hence the LBW fibre type transformation could have contributed to insulin resistance. Nyholm et al. (48) specifically related insulin resistance in relatives of persons with type 2 DM with type IIx fibre proportion. However, the current study indicated no difference in the subgroups of type II fibres, based on MHC assessment. Of note is that the study participants in the Nyholm et al. (48) study had high levels of type IIx fibres (close to 30%) whereas in the African cohort of the current study, the proportion of fast glycolytic fibres indicated by MHC-IIx content was low (less than 10%). Although oxidative enzyme activity, specifically CS activity was related to whole body oxidative capacity in the current study, there were no differences between groups; therefore, the higher circulating glucose levels during the OGTT cannot be explained by low capacity of *m*. vastus lateralis to oxidise carbohydrates. It has previously been shown that fibre type proportions of *m*. vastus lateralis biopsy samples were not correlated to insulin-stimulated glucose uptake in obese persons with low glucose tolerance (49). Although, insulin-stimulated glucose uptake was lower in all muscle groups tested, it was significantly greater for *m*. erector spinae than *m*. rectus abdominis, which are predominantly higher in type I fibres or type II fibres, respectively. Hence, correlations between glucose dynamics and proportion of fibre types in muscles known to have mixed fibre type (of which *m*. vastus lateralis is a frequently assessed example), is most likely less sensitive than when muscles high in a particular fibre type are assessed either in rats or humans. This suggested explanation for our results and those of Koh et al. (49) may be overcome by higher number of study participants.

Nevertheless, our OGTT-stimulated plasma glucose results show that the PPM+ group had an abnormal glucose metabolism. The reduced disposition index indicates that compromised insulin secretion is the main factor behind the increased glucose levels. However, as disposition index is calculated via adjustment for insulin resistance, the reduced disposition index cannot *a priori* exclude the possibility of reduced insulin sensitivity either at hepatic or skeletal muscle level. Hepatic insulin resistance is partly included in the homoeostasis model assessment of insulin resistance, but sensitivity is not reduced in the PPM+ group. However, disposition index does not adjust for peripheral insulin resistance in the skeletal muscle. In brief, the data we present do not indicate insulin resistance in the skeletal muscle, which supports that impaired insulin secretion rather than insulin resistance, explains the mild elevations of plasma glucose levels in the PPM+ group.

Apart from an effect on blood flow from the placenta causing compromised oxygen supply to the foetus, malaria infection has also been shown to affect the microvasculature of several organs of the host (50, 51), here the pregnant woman. This causes metabolic stress, and potentially hypoglycaemia (52), which may compromise nutrient supply to the foetus. However, as the baseline study on malaria prophylaxis was not designed to address metabolic consequences of the pregnant women, blood sugar was not measured, and we remain with the birth weight of the offspring as a proxy for compromised intrauterine growth. Furthermore, birth weight was not compromised in the PPM+ group compared to the two other groups, even if the proportion of LBW had a tendency to be higher. Nevertheless, we have recently shown that insulin levels were lower in early pregnancy anaemia-exposed umbilical cord blood, supporting the notion of developmental programming of pancreatic beta-cell dysfunction and subsequently increased risk of type 2 DM in offspring of mothers with early pregnancy anaemia (53).

We acknowledge several limitations to the current study. First, malaria prophylaxis/suppression may have blunted adverse physiological effects of placental and peripheral malaria infection. As the biopsies were taken in a fasting and not in a stimulated state, we may have missed the detection of higher response related to enzyme activity and thereby differences between groups. Importantly, the study may have been underpowered by small sample sizes, which may have resulted in a statistical type II error. Finally, placental malaria infection changes persist endlessly. Whereas placental swab (thick blood smear) detects active infection, past infection is detected through histopathological changes (54). Unfortunately, histopathological examination results for this study group could not be backtracked, which is an analytical limitation as some past infected placenta may have been missed.

In conclusion, skeletal muscle fibre types or enzymatic activities were not different across groups. Mean fasting and OGTT-derived glucose levels were higher in the group exposed to foetal peripheral and placental malaria and detection of hyperglycaemia as well as low C-peptide levels at the individual level were all from the peripheral and placental malaria exposed group. Compromised insulin secretion rather than insulin resistance seems to be the primary defect, but this needs to be determined in larger and/or methodologically more sophisticated studies.

## Data availability statement

The raw data supporting the conclusions of this article will be made available by the authors, without undue reservation.

## Ethics statement

The studies involving human participants were reviewed and approved by National Institute of Medical Research (NIMR/HQ/R.8a/vol.IX/916). The patients/participants provided their written informed consent to participate in this study.

## Author contributions

DC, IB, TM, KR, AV, and KM: contributors conceptualization. DC, AV, JN, and TM: data collection. DC and KM: formal analysis. DC, AV, and KM: writing (original draft). DC, TM, IB, AV, LG, FL-T, JN, CS, KR, and KM: writing (review and editing). DC and KM take full responsibility for the work. All authors contributed to the article and approved the submitted version.

## Funding

The study was funded by Thorvald Madsen’s Foundation for the Advancement of Medical Sciences and Brdr Hartmann’s Foundation.

## Conflict of interest

DC has received payment from Novo Nordisk Mexico for consultancy work. AV is a shareholder in Astra Zeneca.

The remaining authors declare that the research was conducted in the absence of any commercial or financial relationships that could be construed as a potential conflict of interest.

## Publisher’s note

All claims expressed in this article are solely those of the authors and do not necessarily represent those of their affiliated organizations, or those of the publisher, the editors and the reviewers. Any product that may be evaluated in this article, or claim that may be made by its manufacturer, is not guaranteed or endorsed by the publisher.
