# Supplementary material for: Skeletal muscle fibre type and enzymatic activity in adult offspring following placental and peripheral malaria exposure in foetal life
Source: Front Public Health. 2023 Jun 2;11:1122393. doi: 10.3389/fpubh.2023.1122393 (PMC10275361; doi:10.3389/fpubh.2023.1122393)
Supplement: Supplementary file 1 [file Table_1.docx]

|  | Malaria negative | Peripheral malaria | Peripheral+placental malaria | P-value | P-value adjusted^1^ |
| --- | --- | --- | --- | --- | --- |
| N | 13 | 28 | 9 |  |  |
| SBP (mmHg)^2^ | 123 (15) | 129 (11) | 124 (15) | 0.36 | 0.65 |
| DBP (mmHg)^3^ | 76 (8) | 79 (7) | 75 (11) | 0.29 | 0.84 |
| Resting heart rate (bts/min) | 73 (13) | 79 (14) | 73 (10) | 0.36 | 0.65 |
| Total cholesterol (mmol/L) | 3.5 (0.8) | 3.8 (0.8) | 3.6 (0.6) | 0.10 | 0.54 |
| LDL-C(mmol/L)^4^ | 2.0 (0.5) | 2.1 (0.7) | 1.6 (0.4) | 0.23 | 0.46 |
| HDL-C (mmol/L)^5^ | 1.0 (0.3) | 1.0 (0.3) | 0.9 (0.2) | 0.67 | 0.45 |
| Triglycerides (mmol/L) | 0.7 (0.1) | 0.9 (0.4) | 0.8 (0.2) | 0.30 | 0.42 |
| hs-CRP (mg/L)^6^ | 0.5 (0.3;0.7) | 0.5 (0.3;0.9) | 1.5 (0.2;2.8) | 0.70 | 0.30 |
| ALAT (IU/L)^7^ | 16.0 (4.2) | 14.6 (4.6) | 18.8 (7.1) | 0.15 | 0.45 |
| ASAT (IU/L)^8^ | 33.5 (14.0) | 30.8 (10.2) | 33.5 (8.6) | 0.73 | 0.74 |
| GGT (IU/L)^9^ | 16.6 (7.5) | 16.7 (8.0) | 27.4 (9.1)^a,b^ | 0.01 | 0.026 |
| Carbamide (mmol/L) | 2.3 (0.5) | 2.8 (0.6)^a^ | 2.8 (0.4)^a^ | 0.02 | 0.021 |
| Albumin (g/L) | 41 (2.4) | 41 (2.5) | 39 (7.0) | 0.70 | 0.47 |
| Creatinine (µmol/L) | 70 (10.9) | 69 (13.1) | 72 (6.4) | 0.83 | 0.81 |
| Fatty liver index^10^ | 4.5 (4.6) | 4.9 (4.1) | 9.0 (10.3) | 0.26 | 0.19 |

**Supplemental Table.** Clinical characteristics in offspring stratified by malaria exposure during pregnancy presented as mean (SD) or median (IQR) (n=50)

^1^Adjusted for myosin heavy chain-I percentage; ^2^SBP, Systolic blood pressure; ^3^DBP, Diastolic blood pressure; ^4^LDL-C, low-density lipoprotein cholesterol; ^5^HDL-C, high-density lipoprotein cholesterol; ^6^hs-CRP, high-sensitivity C-reactive protein; ^7^ALAT, Alanine transaminase; ^8^ASAT, Aspartate transaminase; ^9^GGT, gamma Glutamyl transferase; ^10^Fatty liver index, formula:

(e ^ 0.953*loge (triglycerides)+0.139*bmi+0.718*loge (ggt)+0.053*waist circumference

-15.745)/(1 + e ^ 0.953*ln(triglycerides)+0.139*bmi+0.718*loge (ggt)+0.053*waist circumference -15.745)*

100
